# Transverse forces in skeletal muscle with massage-like loading in a rabbit model

**DOI:** 10.1186/1472-6882-14-393

**Published:** 2014-10-13

**Authors:** Thomas M Best, Scott K Crawford, Caroline Haas, Lawrence Charles, Yi Zhao

**Affiliations:** Division of Sports Medicine, Department of Family Medicine, The Ohio State University, Columbus, Ohio USA; Department of Biomedical Engineering, The Ohio State University, Columbus, Ohio USA; Sports Health and Performance Institute, The Ohio State University, Columbus, Ohio USA

**Keywords:** Massage, Muscle, Injury, Eccentric exercise

## Abstract

**Background:**

The objective of this study was to quantify the transverse forces in skeletal muscle subjected to constant compressive massage-like loading (MLL) following eccentric exercise (ECC).

**Methods:**

Twenty-eight New Zealand White rabbits were used for this two-part study. For all testing, a customized electromechanical device was utilized to apply a constant compressive force MLL to the tibialis anterior (TA) muscle and the resultant transverse forces were quantified. The device consisted of two stepper motors that were positioned orthogonally to each other and connected to separate sliding tracks. A stainless steel cylindrical massage tip was mounted to a customized two-axis sensor consisting of two strain gauges with which forces along the two axes were measured. First, we determined the effects of tissue loading frequency and compression magnitude on transverse forces in the TA. Following a bout of ECC, sixteen rabbits were randomly assigned to a protocol with MLL frequency of 0.25 Hz or 0.5 Hz at a constant compressive force of 5 N or 10 N. Secondly, we utilized a protocol of 0.5 Hz, 10 N, 15 min MLL that was performed on 4 consecutive days commencing immediately post ECC (n = 6 animals) or 48 hours following ECC (n = 6 animals). Transverse forces were measured during all 4 MLL sessions for the entire 15 min duration for both the immediate and the delayed groups.

**Results:**

Both frequency and magnitude of compressive force due to MLL showed an effect on the magnitude of transverse force (p < 0.05 for each parameter). Furthermore, MLL beginning immediately following ECC produced higher transverse forces than MLL delayed by 48 hours with an average 20% difference between the two MLL groups over the four day protocol. Forces were higher in the middle 5 minutes compared to the first 5 minutes for all MLL bouts in both groups.

**Conclusions:**

Frequency and magnitude of MLL and timing for delivery of MLL following ECC affect resultant transverse force values for exercised muscle. The application of our findings to humans receiving massage following exercise remains unknown at this time.

## Background

Among the complementary and alternative medicine modalities, massage-based therapies are one of the most frequently used worldwide. In fact, in the United States alone, it was estimated that up to 8% of the population used massage therapies in 2007 [1]. A 2010 conference highlighted the state of affairs of massage therapies and emphasized the need for a better mechanistic understanding of this preventative and restorative treatment, as well as the development of animal models for clinical conditions that resemble conditions massage therapists treat [2].

Studies have claimed that up to 45% of athletes utilized the services of massage therapists during a large sporting event [3]. Several recent reports have suggested that massage-based therapies can modulate the tissue’s inflammatory and immune responses [4–6]. Crane and colleagues [7] studied the effect of a 10 minute bout of massage on 11 young males following eccentric exercise. Muscle biopsies revealed that massage activated the mechanotransduction signaling pathways focal adhesion kinase (FAK) and extracellular signal-regulated kinase ½ (ERK1/2) while at the same time mitigating the increase in nuclear factor қB (NFқB) caused by exercise-induced trauma. Moreover, the 10 minute bout of massage attenuated the production of the pro-inflammatory cytokines tumor necrosis factor-α (TNF-α) and interleukin-6 (IL-6). Similarly, Rapaport and colleagues [8] compared a 45 minute Swedish massage to a light touch control in 53 adults and showed that Swedish massage caused a large effect size decrease in arginine-vasopressin (AVP) and a small effect size in serum cortisol (CORT). Blood levels of IL-1β, IL-4, IL-6, IL-10, and IL-13 were also decreased in subjects receiving Swedish massage compared to touch control. It was concluded that a single session of Swedish massage may have promise for managing inflammatory and autoimmune conditions. Collectively, these studies have begun to address biologic mechanisms for massage-based therapies. However, the forces applied to the tissues during the massage sessions were not quantified making it difficult to establish true causation between the forces and biologic responses.

Although consensus for the clinical indications and prescription for massage therapies and the specific techniques is still rather elusive, a combination of compressive and transverse (longitudinally along the tissue) forces are typically applied [9]. Both superficial and deep techniques are commonly employed, yet the actual force magnitudes applied to the soft tissues (skin, fascia, muscle, tendon, and ligament) are still unknown. To this end, our laboratory has developed an animal model where the mechanical and biologic effects of massage-like loading (MLL) are studied [4, 5, 10, 11]. We attempt to approximate Swedish massage by externally applying a constant compressive force to the rabbit tibialis anterior (TA) muscle following eccentric exercise (ECC) [12, 13]. We have shown that a dose–response relationship exists with an optimal magnitude, duration, and frequency of MLL producing maximal recovery of muscle and joint function [11]. Furthermore, following a bout of severe ECC, 4 days of MLL led to a reduction in tissue passive stiffness on both a daily and cumulative effect, although the additive effect diminishes with time [10]. To our knowledge, these findings provide the first objective and quantifiable measures of change in both active and passive tissue properties with the application of *ex vivo* tissue loading intended to simulate massage. In the current study, we report for the first time on the transverse forces that result under the conditions described above. The purpose of the current study is twofold: 1) to investigate the effects of MLL magnitude and frequency on transverse force values and 2) to compare the effects of immediate versus delayed MLL on transverse forces as a function of time following a controlled bout of ECC. These data should provide a starting point to understand the role of the various forces (compression and transverse) that are produced during certain types of massage and their effects on tissue properties.

## Methods

All experiments were approved by the Institutional Animal Care and Use Committee (IACUC) at The Ohio State University.

### Eccentric exercise

For all reported procedures, surgeries, exercise, and bouts of massage, anesthesia was initiated with 0.25 ml acepromazine via an inner ear vein followed by maintenance with 1.5% isoflourane. One week prior to ECC, animals were surgically implanted with bilateral peroneal nerve cuffs [5, 14] and subcutaneous interfaces for reproducible and repeatable external stimulation of the TA muscle [4, 5, 10, 11]. Seven days after surgery, the animals underwent a damaging bout of ECC.

External connections were made to the interfaces for the nerve cuffs using silicone insulated wire. The ends of the wire were stripped and then threaded through a 22-gauage needle for insertion through the skin of the rabbit into the interfaces. A baseline α-motoneuron threshold voltage was obtained for each animal. The rabbit was secured supine in a sling with the knee joint held at 90 degrees (to the hip) and one foot placed on a foot pedal attached to a ServoMotor with the ankle held at 90 degrees (to the knee) [4].

For both Part 1 and Part 2 of the study, the same ECC protocol was utilized. One hindlimb was subjected to an ECC bout of seven sets of 10 lengthening contractions with 2 min rest periods between sets. For each contraction (external electrical stimulation at three times the α-motoneuron threshold), the ankle was moved within a tibiotarsal angle of 95° to 145° of plantarflexion at 150°s^−1^. Muscle activation preceded stretch of the TA muscle-tendon unit by 100 ms (total stimulus train duration = 433 ms). The ankle was then passively returned by the motor at a velocity of 150°s^−1^ to the initial tibiotarsal angle of 95°.This has proven to be a consistent and repeatable method of inducing injury as measured by reduction in peak isometric torque [4, 5, 11]. In the current study, average reductions in peak isometric torque were 61.8% (±2.1%) in animals used in Part 1and 49.5% (±5.0%) in animals used in Part 2.

### Massage device

**Figure 1:**
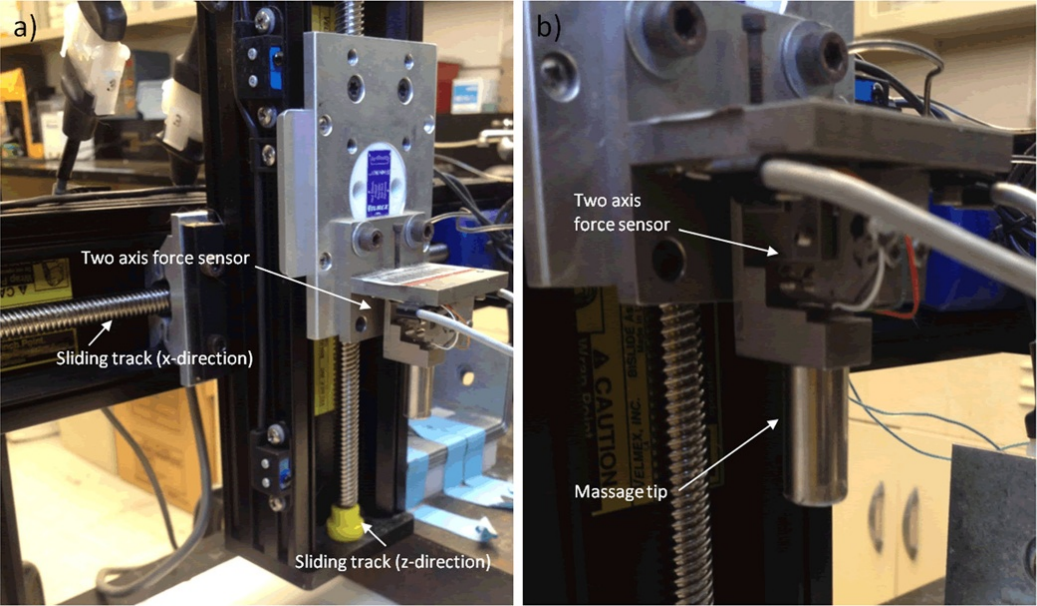
**Massage device. a)** The massage device had two stepper motors mounted on two bi-directional sliding tracks positioned perpendicular to each other. The compressive forces were related to the z-direction, while the transverse forces were related to the x-direction. **b)** The massage tip was mounted on a custom designed two-axis force sensor which consisted of two strain gauges. A known gravitational force of a standard weight was used to determine the relationship between the strain and measured voltage.

The massage device is detailed in Wang et al. [13] and consists of two stepper motors positioned perpendicularly to each other (Figure 1a). Each motor was connected to a bi-directional sliding track with one positioned horizontally (x-axis) and the other positioned vertically (z-axis). The compressive forces were related to the z-axis, while the transverse forces were related to the x-axis. The stepper motors were controlled by the computer, which was also used for data acquisition, in order to control the parameters associated with massage.

A customized two-axis force sensor consisting of two strain gauges was utilized to measure the strain in both directions (x-axis and z-axis). Each gauge had four piezoresistors configured into a Wheatstone bridge. A stainless steel cylindrical massage tip (1/2 in. diameter) was mounted onto the force sensor (Figure 1b). A known gravitational force of a standard weight was used to determine the relationship between the strain and the measured voltage [13]. The travelling stroke and frequency of the motor in the x-direction was controlled using the Cosmos 3.1.0 program. A graphic user interface (GUI) allowed for easy visualization of the applied forces.

**Figure 2:**
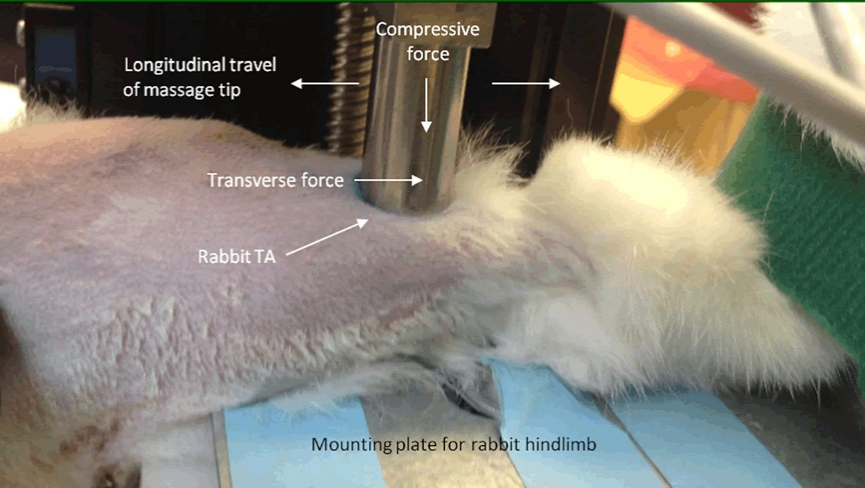
**Setup of massage-** **like loading** **(MLL)** **of rabbit tibialis anterior** **(TA).** While the animal was anesthetized, the foot was strapped onto mounting plate during massage. The massage tip was manually lowered until the desired compressive force (5 N or 10 N) was obtained. MLL was applied to the mid-belly of the TA with strokes applied longitudinally along the muscle belly (in the direction of the muscle fibers).

While the animal was anesthetized, the foot was strapped onto an L-angled mounting plate for each massage bout (Figure 2). The massage tip was manually lowered along the z-axis until the desired compressive force (5 N or 10 N) was obtained and verified in the GUI. The measured forces were fed back by a feedback mechanism, which could be toggled on and off using the GUI, to ensure a consistent and constant compressive force. MLL was administered to the mid-belly of the TA (Figure 2) with strokes applied longitudinally along the muscle belly (in the direction of the muscle fibers) mimicking the effleurage technique of clinically-utilized massage therapies.

### Part 1

In Part 1 of the study, we investigated the effects of compressive forces and velocity of massage strokes on the resultant transverse forces in MLL. Sixteen New Zealand White rabbits (mass 3.72 ± 0.34 kg) were used in the first part of this study. Following ECC, rabbits were randomly assigned to a protocol with MLL frequency of 0.25 Hz or 0.5 Hz (traveling velocity of 3.1 mm/s and 6.2 mm/s, respectively) at a constant compressive force of 5 N or 10 N. This design allowed an n = 4 for each frequency and compressive force combination in order to detect any differences in resultant transverse forces between the various conditions. Prior to MLL, the skin overlying the TA was shaved and no lubricant was used during the MLL bouts. A 15 min bout of MLL was applied for four consecutive days immediately following exercise and occurring daily (24 hours apart).

### Part 2

Here we investigated the effects of applying MLL at different time points following ECC on the transverse forces in the muscle. Twelve New Zealand White rabbits (mass 3.66 ± 0.27 kg) were used in the second part of the study. One leg was randomly selected to undergo the ECC protocol while the other limb served as an unexercised control. The 12 animals were then randomized to receive MLL immediately following the bout of exercise (n = 6) or beginning 48 hours after the exercise (n = 6). The MLL protocol (0.5 Hz, 10 N, 15 min) [11] was carried out over 4 consecutive days. Transverse forces were quantified during all 4 bouts of MLL for all 12 animals using the previously described customized device (Figure 1) [13].

### Statistical methods

For both Parts 1 and 2, we measured the resultant transverse forces over the entire 15 min for each bout of MLL. Preliminary analysis showed less than 2% variation in these forces across each of the 5 min intervals. Therefore, we choose the middle ten loading cycles to calculate the resultant average transverse forces. Thus, for Part 1 each animal for each group (n = 4) was able to supply 10 measurements of transverse force magnitudes for each of the 4 days of MLL resulting in a total of n = 40 for each animal. As a result, there was an n = 160 for each combination of frequency and compressive force (0.25 Hz and 5 N; 0.25 Hz and 10 N; 0.5 Hz and 5 N; and 0.5 Hz and 10 N). This sample provided statistical power above 0.8 for a significance level of 0.05 for both of the main effects of frequency and compressive magnitude. Additionally, we calculated a power of 0.515 for the interaction with a least significant number of measurements in order to detect a significant difference due to an interaction of 579. Thus, as determined by our statistical analysis, we have enough samples to detect differences in shear force magnitudes due to both main effects and their interactions.

For Part 1, ANOVA was performed to detect differences of each parameter on shear force magnitudes and their interaction. Tukey-Kramer tests were performed to detect differences among all pairs of interactions. For Part 2, ANOVA was performed to detect differences in shear force magnitudes between groups, MLL bout (Days 1–4) within group, and any interactions. Tukey-Kramer tests were also performed to compare all pairs of treatments. Each variable was modeled as discrete and nominal. All data were normally distributed as determined by the Shapiro-Wilk test. Significance level was set at α = 0.05 for all comparisons. JMP 10.0.2 (SAS Institute Inc, NC, USA) was used for all analysis.

## Results

### Part 1

We wanted to determine if changes in MLL loading frequency and magnitude affected resultant transverse force. Both frequency and compressive force had a significant effect on resultant transverse force (p < 0.001 for both). Additionally, the interaction between the two parameters had a significant effect (p = 0.046). Tukey tests showed that all pairs of the combination of MLL parameters (force and frequency) had a significant effect (p < 0.001 for all) on resultant transverse force, except for the comparison of parameter pairs 0.5 Hz and 10 N vs. 0.25 Hz and 5 N (p = 0.720).

**Figure 3:**
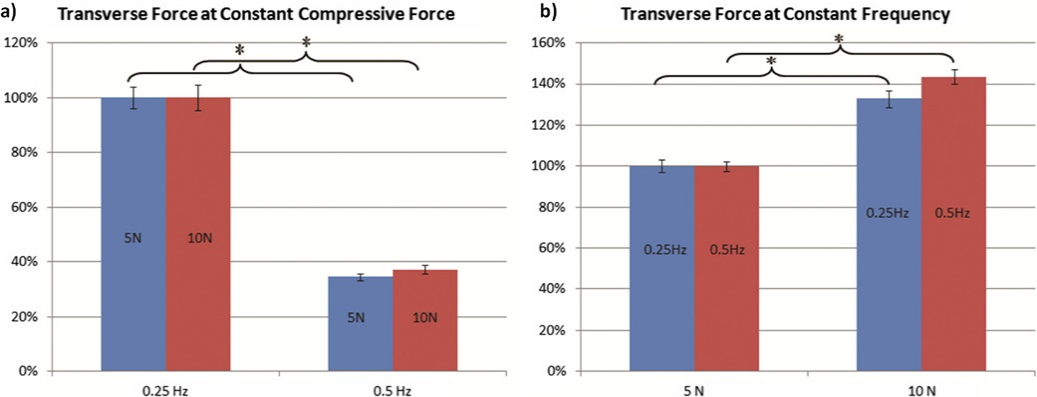
**Resultant transverse force varying loading parameters. a)** Percent change in transverse forces at constant compressive force (5 N and 10 N). Data was normalized by average transverse forces of each compressive level at 0.25 Hz. Note that increasing frequency (traveling velocity of the massage tip) decreased the resultant transverse force at both 5 N (66% decrease) and 10 N (63% decrease) of compressive force. **b)** Percent change in transverse forces at constant compressive frequency (0.25 Hz and 0.5 N). Data was normalized by average transverse forces of each frequency level at 5 N. Note that increasing compressive force increased resultant transverse force at both 0.25 Hz (33% increase) and 0.5 Hz (43% increase).

In order to determine differences in transverse forces due to varying frequency, forces at the two compressive levels (5 N and 10 N) were normalized by the average transverse forces at 0.25 Hz (Figure 3a). The transverse forces at the two frequency levels (0.25 Hz and 0.5 Hz) were normalized by the average transverse forces at 5 N in order to determine differences in transverse forces due to varying compressive force (Figure 3b). Increasing the frequency of tissue loading from 0.25 Hz to 0.5 Hz while keeping compressive force constant at 5 N produced an average 65.9 ± 4.0% decrease in resultant transverse force (Figure 3a). Increasing the frequency of tissue loading from 0.25 Hz to 0.5 Hz while keeping compressive force constant at 10 N produced an average 62.8 ± 4.6% decrease in resultant transverse force (Figure 3a). Increasing compressive force of tissue loading from 5 N to 10 N while keeping frequency constant at 0.25 Hz produced an average 32.9 ± 3.0% increase in resultant transverse force (Figure 3b). Varying compressive force from 5 N to 10 N at a constant frequency of 0.5 Hz resulted in a 43.5 ± 2.4% increase in transverse forces (Figure 3b).

### Part 2

**Figure 4:**
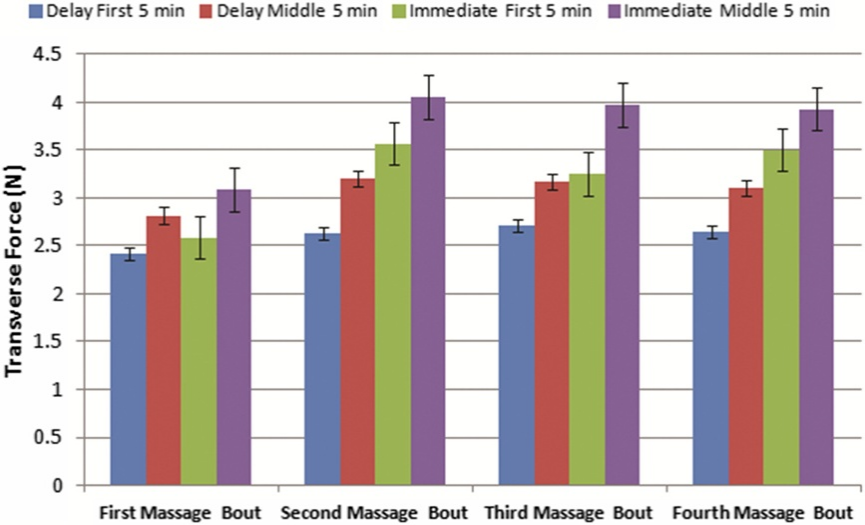
**Immediate and delayed transverse forces for first and middle 5 min of four consecutive days of MLL.** Note an increase in force between the first and middle 5 min on the same day for both groups on all days of MLL.

For both the immediate and delayed MLL groups, average transverse force over the first five minutes was less than that during the middle five minutes (Table 1, Figure 4). Force levels were higher in the immediate massage group compared to the delayed MLL condition. However, there was no significant change (as determined by a Student’s t-test) in average transverse forces between the middle five minutes and the final five minutes (p > 0.05). Percent differences were calculated between groups for the first and middle 5 min of each MLL session (Table 2). There were average 6.8%, 30.4%, 18.5%, and 28.0% differences in force magnitudes between groups in the first 5 min for MLL bouts 1, 2, 3, and 4, respectively. There were average 9.2%, 23.4%, 22.7%, and 23.4% differences in force magnitudes between groups in the middle 5 min for MLL bouts 1, 2, 3, and 4, respectively. The average percent differences across all four days were 20.9% and 19.7% for the first 5 min and middle 5 min, respectively.

**Table 1:** **Average transverse force** (**N**) **for consecutive days of immediate and delayed MLL in the first 5 and middle 5 min of protocol**

	First MLL application		Second MLL application		Third MLL application		Fourth MLL application	
	First 5 min	Mid 5 min	First 5 min	Mid 5 min	First 5 min	Mid 5 min	First 5 min	Mid 5 min
Immediate	2.58 ± 0.17	3.08 ± 0.24	3.56 ± 0.32	4.05 ± 0.37	3.25 ± 0.25	3.97 ± 0.33	3.50 ± 0.25	3.92 ± 0.22
Delay	2.41 ± 0.17	2.81 ± 0.22	2.62 ± 0.17	3.20 ± 0.25	2.70 ± 0.18	3.16 ± 0.15	2.64 ± 0.15	3.10 ± 0.19

(Mean ± standard error).

**Table 2:** **Average percent difference in transverse force between immediate and delayed groups in the first and middle 5 min of daily MLL**

First MLL application		Second MLL application		Third MLL application		Fourth MLL application	
First 5 min	Mid 5 min	First 5 min	Mid 5 min	First 5 min	Mid 5 min	First 5 min	Mid 5 min
6.8%	9.2%	30.4%	23.4%	18.5%	22.7%	28.0%	23.4%

We ran two separate analyses for the first and middle 5 minutes of MLL. ANOVA analysis showed that MLL group (p < 0.0001 for both first and middle 5 min), MLL bout (p < 0.0001 for both first and middle 5 min), and the interaction of day and group (p = 0.0002 for first 5 min, p = 0.0006 for middle 5 min) had a significant effect on transverse forces. Tukey tests showed there was a difference in transverse forces between days 1 and 2, days 1 and 3, and days 1 and 4 in first and middle 5 min (p < 0.0001 for all pairs in first 5 min; p < 0.0001 for all pairs in middle 5 min). There were no differences between days 2 and 3 (p = 0.329), days 3 and 4 (p = 0.496), and days 2 and 4 (p = 0.992) in the first 5 min. There were also no differences between days 2 and 3 (p = 0.892), days 3 and 4 (p = 0.902), and days 2 and 4 (p = 0.499) in the middle 5 min.

**Figure 5:**
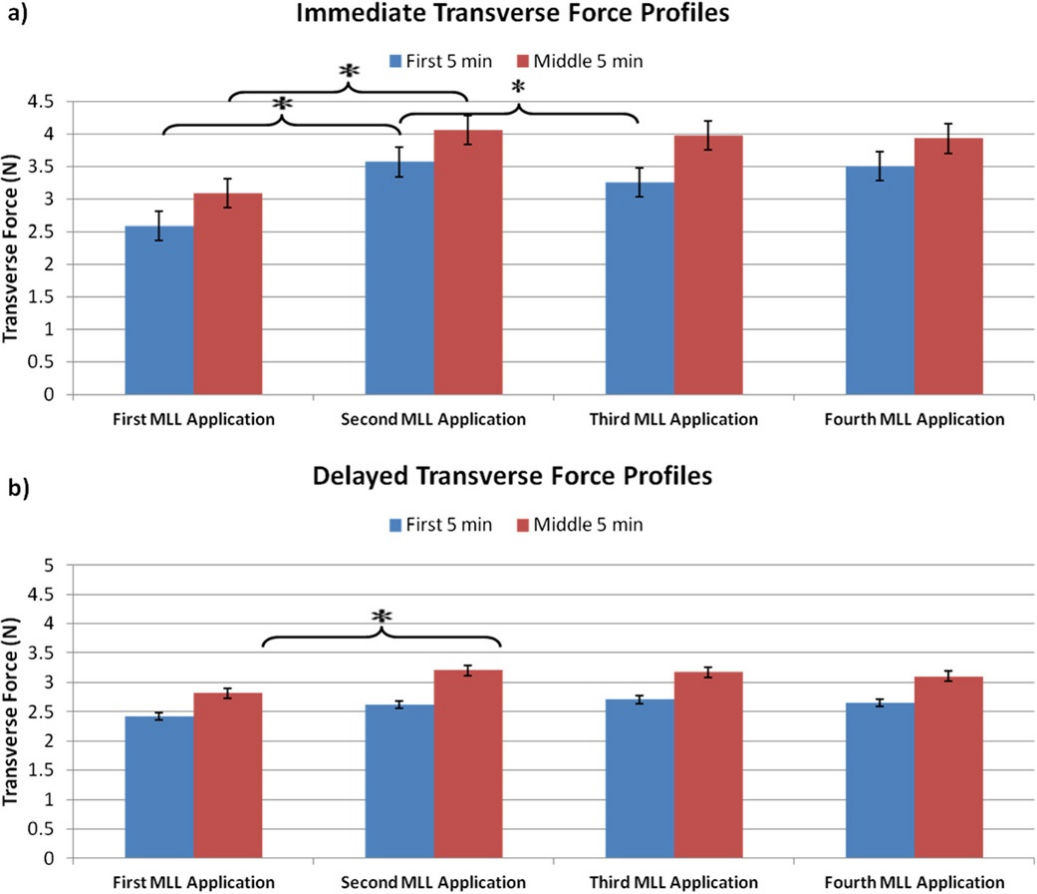
**Transverse force in first and middle 5 min of four consecutive days of MLL** **(0.5 Hz,** **10 N,** **15 min)** **protocol. a)** Immediate MLL. Note there was a difference in transverse force in the first 5 min (p < 0.0001) and middle 5 min (p < 0.0001) between the first and second MLL bouts and a difference in transverse force in the first 5 min between the second and third MLL bouts (p = 0.028). **b)** Delayed MLL. Note there was a difference in transverse force in only the middle 5 min between the first and second bouts of MLL (p = 0.021).

Tukey tests were also used to compare the differences in transverse forces in the first and middle 5 min of MLL between consecutive bouts. As shown in Figure 5a, there was a significant difference in transverse forces during the first 5 min in the immediate group between the first and second massage bout (p < 0.0001) and between the second and third bout (p = 0.028). However, there was no difference in transverse forces in the first 5 min in the immediate group between the third and fourth massage bouts (p = 0.162). There were no significant differences in the transverse forces in the first 5 min in the delayed massage group between any consecutive bouts of MLL (Figure 5b; p = 0.413 between the first and second bouts, p = 0.991 between the second and third bouts, and p = 0.998 between the third and fourth bouts).

Comparisons between consecutive bouts in the middle 5 min of each group were also made (Figure 5). There was a significant difference in transverse forces in the middle five minutes between the first and second massage bouts in both immediate (p < 0.0001) and delayed groups (p = 0.0213) as shown in Figure 5a and 5b, respectively. There were no differences in average transverse forces in the middle five minutes between the second and third (p = 0.996 for immediate and p = 1.000 for delayed groups) or third and fourth massage bouts for both groups (p = 0.999 and p = 0.992 for immediate and delayed groups, respectively).

## Discussion

The main finding of the current study is that during constant compression MLL (intended to simulate massage) transverse forces in the tissue are dependent on both loading frequency (traveling velocity of the massage tip) and compressive force magnitude. Additionally, we observed that MLL applied to the muscle immediately following ECC produced greater transverse forces along the tissue than delayed MLL and that these forces were highest in the middle five minutes of a 15 min MLL session.

It has been suggested that physiological benefits of massage are likely to be initiated through mechanical effects on tissues followed by changes in intracellular regulatory pathways [7]. Therefore, the mechanical signals leading to changes in intracellular regulatory pathways likely influence gene expression, protein synthesis, and muscle metabolism. Modifications in the magnitude, frequency, and duration of mechanical loading due to massage have indeed been shown to influence recovery of muscle active and passive properties following eccentric exercise [10, 11]. However, the translation of these findings from preclinical models to clinical practice has been challenging. This is likely due to a number of factors including: a lack of understanding of the magnitude of the forces applied during manual therapies [15]; an incomplete understanding of the physiologic window for biologic tissues that defines the catabolic-anabolic homeostasis [16]; and an increasingly clearer understanding that these responses may be highly individualized.

Many studies have investigated the effects of compressive forces of massage on the recovery of muscle active properties, pain reduction, and tissue inflammation [5, 6, 11, 17]. To our knowledge, this is the first study to quantify transverse forces during constant compressive MLL. In Part 1, we observed that the transverse forces were dependent upon both frequency and magnitude of tissue loading. When increasing the compressive force of MLL, we observed a 33% increase in transverse force at a frequency of 0.25 Hz and a 43% increase in transverse force at a frequency of 0.5 Hz. This finding is consistent with the fundamental knowledge of factors affecting frictional forces (in biologic tissues). Friction is a function of both normal (compressive) force and the coefficients of friction—the amount of resistance to the initiation of movement (static frictional coefficient) or the resistance to movement (dynamic coefficient of friction). As the compressive force increases, the frictional force increases thus increasing the transverse forces on the tissues.

Frequency, or the traveling velocity of the massage tip, also had a significant effect on the resultant transverse forces. We observed a 66% decrease in transverse force at a constant compressive force of 5 N and a 63% decrease at 10 N when the traveling velocity of the mechanical device (longitudinally along the muscle belly) was increased. We hypothesize that this decrease in transverse forces could be due to the viscoelastic properties of the muscle. As the massage tip travels along the muscle belly, areas not subjected to the compressive load recover slightly from its deformation. The amount of recovery depends on the traveling velocity of the massage tip, with a greater amount of recovery from its compressed state occurring at lower velocities (i.e. more time to recover to its original, undeformed state). This subsequently results in more tissue to envelop the massage tip, thus increasing the amount of resistance on the tip as it travels along the muscle belly. An alternative theory is that fluid dissipates more quickly when subjected to higher traveling velocities of massage. The greater amount of fluid dissipation would result in decreased resistance on the massage tip and a subsequent decrease in the transverse forces. It is not known from the current study however if the observed transverse forces are due to fluid movement or tissue resistance itself. Moreover, recent studies using elastography have shown an increase in passive muscle stiffness in the first hour following eccentric exercise which could account, in part, for the changes in transverse forces we attribute to MLL in the current study [18, 19].

In Part 2, we compared immediate and delayed MLL and their effects on transverse forces in the muscle. We observed that transverse forces were higher in the second five minutes compared to the first five minutes of the 15 min MLL application. As noted above, this finding could be in part related to the change in passive tissue stiffness in the early period following ECC [18, 19]. We hypothesize that the relaxation of the muscle during the first five minutes resulted in the increase in transverse forces during the second five minutes of MLL. Due to the muscle’s viscoelastic behavior, the muscle tissue relaxed during the first five minutes due to mechanical adaptation of the muscle to the compressive load, thus reducing the amount of force exerted on the muscle. This is clinically significant because, as noted previously in Part 1 of the study, it appears that transverse tissue forces associated with massage are dependent on frequency and compression magnitude. Additionally, it is known that increased transverse force is accompanied by increased shear strain. As observed *in vitro* [20], an increased release of bFGF occurred at larger amounts of shear strain suggesting that the changes to intracellular regulatory pathways are dependent on the amount of shear strain applied to the cells. The muscle’s relaxation would result in decreased shear strain, perhaps reducing the release of these growth factors and cytokines.

We also observed that MLL started immediately following exercise resulted in larger transverse forces than MLL delayed by 48 hours. We propose this finding is due to the difference in the number of muscle fibers that resist mechanical loading between the two groups. In our previous work, we observed that immediate MLL was more effective in limiting damage (torn muscle fibers) than the same protocol delayed by 48 hours [5]. Therefore, due to the increased number of muscle fibers theoretically still in continuity in the immediate group and the subsequent resistance of these fibers to applied forces, immediate massage resulted in larger transverse forces compared to delayed massage.

In the clinical setting, many different massage techniques are used, with each approach desiring specific outcomes through various combinations of compressive and transverse forces. To our knowledge, the forces associated with massage therapies have not yet been quantified in humans. We acknowledge that our model has several limitations including the requirement for anesthesia and a test system that, at best, approximates the application of massage to humans. For example, Swedish massage uses a combination of long strokes, kneading, deep circular movements, vibration and tapping [9]. Our device has the capacity to model many of these techniques but only as individual motions. Furthermore, the effects of the anesthesia on our results and how this would apply to humans is difficult to ascertain from our studies. Nevertheless, the current study provides a basis to understand the resultant transverse forces, and in conjunction with our previous work, may provide a starting point in identifying the optimal time course and tissue loading parameters, their effects on tissue properties, and subsequent biological responses that may be possible using massage-based therapies in humans.

## Conclusions

To our knowledge, this is the first study to quantify the transverse forces associated with muscle MLL. We observed that transverse forces were dependent upon the frequency (traveling velocity of the massage tip) and the magnitude of muscle compression. Additionally we observed a time-dependent relationship of the transverse forces within individual MLL sessions and when MLL was applied following the exercise. In conjunction with previous studies, these data provide a starting point for identifying optimal loading parameters that could be used during manual therapies, optimal time courses for their application, and the effects of manual therapies such as massage on the tissues’ biological responses.

## Abbreviations

TA: Tibialis anterior
ECC: Eccentric exercise
MLL: Massage-like loading.
